# PRDECT-ID: Indonesian product reviews dataset for emotions classification tasks

**DOI:** 10.1016/j.dib.2022.108554

**Published:** 2022-08-24

**Authors:** Rhio Sutoyo, Said Achmad, Andry Chowanda, Esther Widhi Andangsari, Sani M. Isa

**Affiliations:** a Computer Science Department, School of Computer Science, Bina Nusantara University, Jakarta 11480 Indonesia; b Psychology Department, Faculty of Humanities, Bina Nusantara University, Jakarta 11480 Indonesia; c Computer Science Department, BINUS Graduate Program - Master of Computer Science, Bina Nusantara University, Jakarta 11480 Indonesia

**Keywords:** Natural language processing, Text processing, Text mining, Emotions classification, Sentiment analysis

## Abstract

Recognizing emotions is vital in communication. Emotions convey additional meanings to the communication process. Nowadays, people can communicate their emotions on many platforms; one is the product review. Product reviews in the online platform are an important element that affects customers’ buying decisions. Hence, it is essential to recognize emotions from the product reviews. Emotions recognition from the product reviews can be done automatically using a machine or deep learning algorithm. Dataset can be considered as the fuel to model the recognizer. However, only a limited dataset exists in recognizing emotions from the product reviews, particularly in a local language. This research contributes to the dataset collection of 5400 product reviews in Indonesian. It was carefully curated from various (29) product categories, annotated with five emotions, and verified by an expert in clinical psychology. The dataset supports an innovative process to build automatic emotion classification on product reviews.

**Specifications Table**

**Table:** 

Subject	Computer Science
Specific subject area	Indonesian Language, Natural Language Processing, Text Classification
Type of data	Text Files
How the data were acquired	Information extraction from online marketplace
Data format	Raw
	Analyzed
Description of data collection	Product reviews were extracted from one of the biggest marketplaces in Indonesia, namely Tokopedia. It was collected selectively to ensure distributed data for each data label. There are 5400 product review data that this work annotated with a single emotion label, that is, love, happiness, anger, fear, or sadness.
Data source location	The product reviews were collected at Bina Nusantara University, Indonesia
Data accessibility	Repository name: Mendeley Data
	DOI: 10.17632/574v66hf2v.1[1]
	Direct URL to Data: https://data.mendeley.com/datasets/574v66hf2v/1

## Value of the Data

• To the best of our knowledge, the PRDECT-ID dataset is the first Indonesian product reviews dataset annotated with emotions.
• The PRDECT-ID dataset contains 5400 product reviews, spread over 29 different product categories and ready to use for an emotions classification task.
• The annotation process follows emotions annotation criteria created by an expert in clinical psychology.
• The PRDECT-ID dataset offers additional attributes from the product reviews for other text-classification tasks. For instance, sentiment classification by using the “Sentiment” data.

## 1 Data Description

Generally, the public expresses their opinions via social networking services, such as Twitter [2], [3]. We can also find public opinions on services and products on e-commerce platforms in the form of product reviews [4], [5]. Product reviews are an essential element that affects buying decisions [6].

Tokopedia was founded in 2009 and has become one of the e-commerce giants in Indonesia. Tokopedia’s webpage achieved 147 million views in the second quarter of 2021, while Shopee ranked second with 126 million [7]. Other competitors in e-commerce, namely Blibli, Bukalapak, and Lazada, each reached fewer than 30 million web views.

The PRDECT-ID is a collection of product reviews from Tokopedia. The PRDECT-ID stands for Product Reviews Dataset for Emotions Classification Tasks - Indonesian [1]. There is a total of 5400 product reviews in the PRDECT-ID.

Emotion plays an important role in human language to show a certain perception of a condition or situation. Furthermore, it plays a central role in individual experiences [8], such as the experience of shopping online. In their research [9], Shaver et al. defined five basic-level emotion categories, i.e., love, happiness, anger, fear, or sadness. Each emotion is generally with a lexicon set of words. For instance, the words “shame,” “sympathy,” and “pity” are associated with sadness. Another example is the words “envy,” “hatred,” and “distrust” are associated with anger.

Each product review is annotated with a single emotion with Shaver’s emotions model [9]. Shaver’s emotions model is quite popular as the reference for emotions labeling [10], as it is simple and quite powerful to build a computational emotions model. The annotator label each product review based on the content of the review from the customers. The annotation process follows the emotions annotation criteria created by lecturers and experts in clinical psychology. The annotation criteria is shown in Table 1. Each emotion has a distinctive sentence characteristic. For instance, anger emotion generally contains swearing words and expressing dislike. Moreover, fear emotion contains warning sentences and doubts about the quality of the product or seller.

**Table 1:** Emotions annotation criteria.

Emotions	Sentence characteristic	Sentence examples
Anger	- contains swearing words - express anger - complain and dislike the product/service/delivery - contains punctuation / capital letters - contains words that express annoyed / hate	bad stuff!!! it’s been three days since the edge has been removed, the item is expensive, but the quality is horrible *(barang jelek!!! tiga hari sudah pada lepas pinggirnya, barang mahal tapi kualitasnya jelek banget)*

Fear	- contains warnings sentence - worries about the product - doubt and question the product/seller/delivery	For those of you who want to buy it here, I suggest you make an unboxing video, then turn it on and install CPU Z right away. *(buat agan agan yang mau beli disini, saya cuman bisa saran buat bikin video unboxing, terus hidupin langsung instalin cpu z.)*

Happy	- contains praise - liked the product or expressed satisfaction - contains pride for the product/seller - quality sentences for the product/seller	Excelent. the admin always pays attention to the buyer. Respect, super fast process, arrived quickly too, the item is appropriate, great, thanks *(mantep adminnya selalu merhatiin pembeli. Respect, proses super cepat, sampai jg cepat, barang Sesuai, mantaaaap, thanks)*

Love	- contains feelings or expressions of love - satisfied with the product - contain “excessive” expression / contains hyperbole sentence - contains praise for the product/seller - contains pride for the product/seller	The product is good, and I like it very much!!! *(produknyaaa bagus dan sukaaakkk banggettt!!!)*

Sadness	- express disappointment with the product - express regret towards the product	very disappointed, the phone holder is incomplete, the connector is not there, the packing only uses black plastic *(sangat kecewa, phone holder tidak lengkap penyambung nya tidak ada, packing cuman pake keresek hitam doang)*

With the annotated dataset, the PRDECT-ID is useful for building prediction models to classify the given product reviews automatically. Research conducted by Kusal et al [11] mention that product reviews are quite important since they can determine customer behavior, mindsets and simultaneously give prior information to consumers deciding to buy products. By analyzing the emotions behind product reviews, it is possible to adapt chatbots or conversational agents to improve service quality by giving early feedback and improving market competitiveness. The PRDECT-ID also helpful for other natural language processing related-tasks, such as language generation [4], opinion mining [12], and summarization [13].

Although this work focuses on product reviews, other details related to the product review are captured, such as Price, Number Sold, and Total Review. The authors included these attributes to support further research and combine several product review features. The buyers voluntarily give the product review posted in Tokopedia. Tokopedia’s privacy policy declares that the product reviews provided by buyers are publicly viewable with the consent of the users [14]. Moreover, the dataset protects the buyers’ privacy by ensuring that attributes in the dataset do not include the personal information that identifies or can be used to name Tokopedia users. The list of attribute is shown in Table 2. The extracted data represents an existing attribute, and the description of each attribute is explained in the Description column. Price, Overall Rating, Number Sold, and Total Review are included to help understand the reviewed product better, so the researcher can explore what product has the most “anger” review, how much the price, and hows the overall ratings of the product. The Sentiment and Emotion are label data from the result of annotations, so this data supports both sentiment analysis and emotion classification. This work also collects the seller’s name and the product’s link. Nevertheless, those data are not shown and shared because of privacy concerns.

**Table 2:** List of attributes for the data extraction.

Attribute	Description
Category	Product classification by category
Product Name	Name of the reviewed product
Location	City name of the shop or product seller
Price	Price in IDR of the reviewed product
Overall Rating	Overall product rating
Number Sold	Total number of products sold
Total Reviews	Total number of reviews given by the customers
Customer Rating	Product rating (range 1 to 5) from the customers
Customer Review	Product reviews given to the product by the customers
Sentiment	Sentiment labels (i.e., Positive, Negative)
Emotion	Emotion labels (i.e., Anger, Fear, Happy, Love, Sadness)

**Fig. 1:**
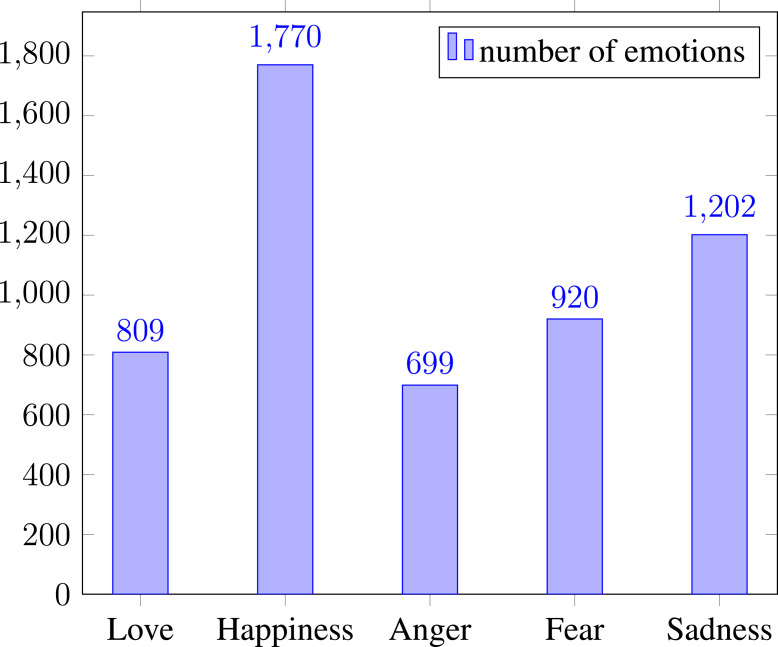
Emotions distribution in the PRDECT-ID dataset.

The dataset of PRDECT-ID is stored in a single spreadsheet (.csv) file. It can be accessed via Mendeley Data for academic and research purposes [1]. Each row of the datasets contains emotion labels and sentiment labels. Fig. 1 shows the distribution of emotions in the PRDECT-ID dataset. In total, there are 5400 product reviews. The happy emotions are the emotions with the most significant number, followed by the sadness emotions in second. In Indonesian product reviews, it is easier to find happiness and sadness emotions since the buyer could be satisfied or disappointed with the product, services, or delivery. On the other hand, the emotion of love and anger is infrequent in buyers’ reviews. Based on Shaver’s emotion model, love and happiness are positive emotions. Furthermore, anger, fear, and sadness are negative emotions [9]. Thus, there are 2579 positive emotions and 2821 negative emotions. This work finds that product reviews with four ratings usually contain mixed emotions during the product reviews’ search. This work tries to avoid those data because it is ambiguous. The dataset is quite imbalanced and might cause problems in modeling the emotions classification task. However, it can be solved using several methods, such as: data augmentation and sampling, as well as, adjusting weights distribution for each class. We argue that the dataset should represents the real-world situation, where most of the datasets are imbalanced.

**Fig. 2:**
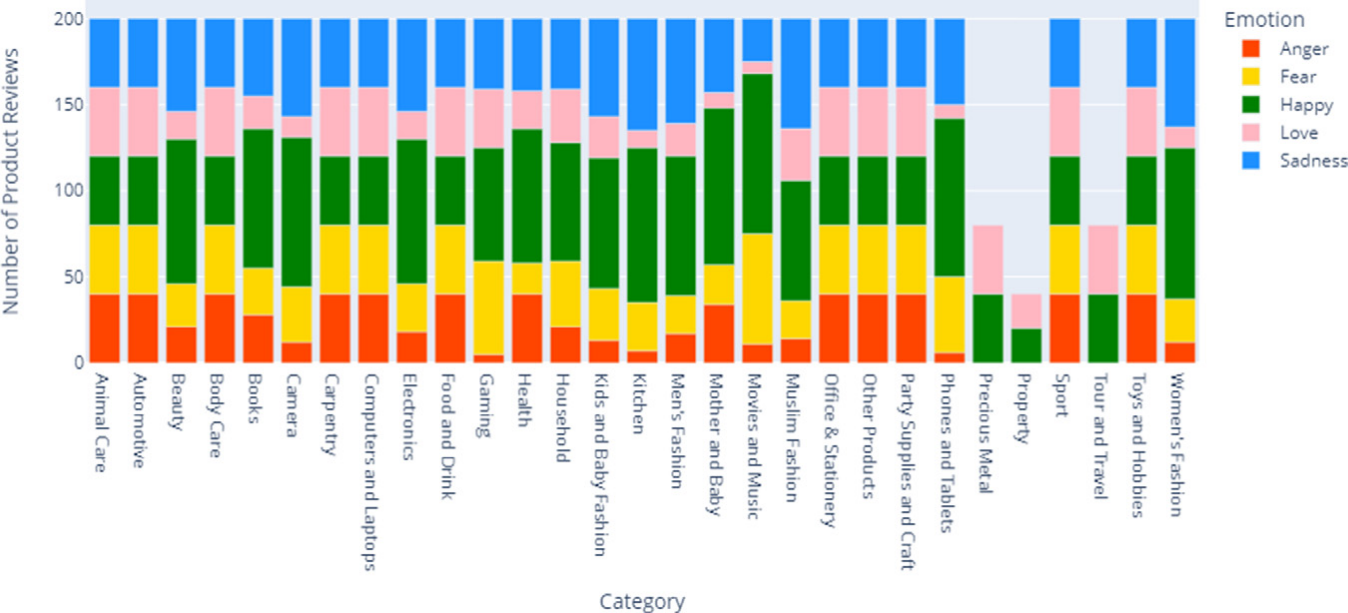
Distribution of categories and their emotions labels.

The distribution of categories and their emotions labels is shown in Fig. 2. The product reviews are selected based on the annotation criteria shown in Table 1. Ten product categories have an even distribution of emotions for all emotion labels, with 40 reviews per emotion label. The categories are Animal Care, Automotive, Body Care, Carpentry, Computers and Laptops, Food and Drink, Office and Stationery, Party Supplies and Craft, Sport, and Other Products. Meanwhile, in other categories, such as Precious Metal, Property, and Tour and Travel, there were no expressions of the emotions of sadness, fear, and anger at all. The distribution of emotional labels in each category depends on the number of products purchased. Product reviews will find more diverse emotional expressions for categories with many buyers.

As of April 2022, there are 30 categories in Tokopedia. The wedding category does not have buyers; thus, the PRDECT-ID dataset can only extract product review data from 29 categories. Moreover, some categories do not contain several emotions, i.e., the category of precious metal, property, and tour & travel. The product reviews in the property category generally contain only ratings; there is no text review. The love emotion is generally detected in product reviews with five ratings. The happy emotion is discovered in product reviews with four to five ratings. The sadness and fear are found in product reviews with one to three ratings. Lastly, anger emotion is usually detected in product reviews with one to two ratings. The number of each emotion by category is shown in Table 3.

**Table 3:** Distribution of categories and their emotions label.

Categories	Anger	Fear	Happy	Love	Sadness
Animal Care	40	40	40	40	40
Automotive	40	40	40	40	40
Beauty	21	25	84	16	54
Body Care	40	40	40	40	40
Books	28	27	81	19	45
Camera	12	32	87	12	57
Carpentry	40	40	40	40	40
Computers and Laptops	40	40	40	40	40
Electronics	18	28	84	16	54
Food and Drink	40	40	40	40	40
Gaming	5	54	66	34	41
Health	40	18	78	22	42
Household	21	38	69	31	41
Kids and Baby Fashion	13	30	76	24	57
Kitchen	7	28	90	10	65
Men’s Fashion	17	22	81	19	61
Mother and Baby	34	23	91	9	43
Movies and Music	11	64	93	7	25
Muslim Fashion	14	22	70	30	64
Office & Stationery	40	40	40	40	40
Other Products	40	40	40	40	40
Party Supplies and Craft	40	40	40	40	40
Phones and Tablets	6	44	92	8	50
Precious Metal	0	0	40	40	0
Property	0	0	20	20	0
Sport	40	40	40	40	40
Tour and Travel	0	0	40	40	0
Toys and Hobbies	40	40	40	40	40
Women’s Fashion	12	25	88	12	63

Total	699	920	1770	809	1202

## 2 Experimental Design, Materials and Methods

The PRDECT-ID dataset is collected directly through the Tokopedia website. The PRDECT-ID dataset contains product reviews from 29 product categories on Tokopedia that use the Indonesian language. The dataset is collected by accessing each product category on the website, then accessing products with reviews and saving reviews given by buyers. Through the PREDCT-ID dataset, the author tries to provide a comprehensive, publicly available, and ready-to-use product review dataset like the amazon product review dataset [4]. The amazon product review dataset is a collection of product reviews from amazon e-commerce in English. Although it is provided with many attributes related to its products, the amazon product review dataset does not yet have an emotion or sentiment label.

Another product review dataset can be found in the study by Warsito et al. [15]. The dataset name is Tokopedia product reviews. The Tokopedia product review is a product review using the Indonesian language collected from the e-commerce Tokopedia. With the same data source as the PREDCT-ID dataset, Tokopedia product reviews only have sentiment labels obtained through an automatic annotation process using Lexicon-Based. Unfortunately, the dataset is not publicly available. Sun et al. [16] also uses datasets sourced from Tokopedia product reviews. The data collected is millions of product review data from 18 categories on Tokopedia. However, the dataset has neither a sentiment label nor an emotion label, and it is not publicly available.

Since there is no Indonesian product review dataset publicly available and annotated with emotion labels, to provide a comprehensive dataset, the following process is dataset annotation by the group of annotators to provide emotion labels and sentiment labels. There are three annotators in the data collection and data annotation process. Each annotator is assigned different product categories. Emotion and sentiment labels are given to each line of existing product review data, and then the agreement between annotators is determined.

This work plans to gather product reviews from each category of Tokopedia. The target is to extract 40 product reviews of each emotion for each category. Nevertheless, some categories do not have product reviews or specific emotions. The result is 5400 data lines containing product reviews that have emotional and sentiment labels. Furthermore, there are several additional attributes extracted along with the process. The full attributes is shown in Table 2 and the distribution of categories and their emotions label is shown in Table 3. In the data annotation process, this work creates an emotions annotation criteria table shown in Table 1. It was created by an expert in clinical psychology. The annotators then utilized the table to select and annotate each product review extracted from Tokopedia carefully. After the data extraction and data annotation process, the annotators perform peer review to check the quality of the data. Moreover, the authors also perform random data checking to ensure the annotation quality. First, the author ensures that no null value is found in any dataset attribute. Then, uniform the data types for each attribute, specifically for attributes with number types such as Price, Overall Rating, Number Sold, Total Reviews, and Customer Ratings. The writing style was also standardized by not using a thousand separators and a period (.) as a decimal separator. Furthermore, the quote mark (“ ”) was added to the Product Name and Customer Review data.

The data collection and annotation were collected with a spreadsheet program, Microsoft Excel and Google’s Sheets. At the beginning of the data collection, the authors decide what data will be collected and extracted into the result table. The emotion labels are written categorically, i.e., love, happiness, anger, fear, and sadness. Moreover, the sentiment labels are also written similarly, i.e., positive and negative.

## Ethics Statements

The collected data has been fully anonymous and the Tokopedia’s data redistribution policies were complied with [14].

## CRediT authorship contribution statement

**Rhio Sutoyo:** Conceptualization, Methodology, Writing – original draft. **Said Achmad:** Software, Writing – original draft. **Andry Chowanda:** Resources, Writing – review & editing, Funding acquisition. **Esther Widhi Andangsari:** Conceptualization, Validation. **Sani M. Isa:** Validation, Supervision.

## Declaration of Competing Interest

The authors declare that they have no known competing financial interests or personal relationships that could have appeared to influence the work reported in this paper.

## Data Availability

Product Reviews Dataset for Emotions Classification Tasks - Indonesian (PRDECT-ID) Dataset (Original data) (Mendeley Data)
